# IDSM ChemWebRDF: SPARQLing small-molecule datasets

**DOI:** 10.1186/s13321-021-00515-1

**Published:** 2021-05-12

**Authors:** Jakub Galgonek, Jiří Vondrášek

**Affiliations:** Institute of Organic Chemistry and Biochemistry of the CAS, Flemingovo náměstí 2, 166 10 Prague 6, Czech Republic

**Keywords:** Small-molecule datasets, Resource Descriptor Framework, SPARQL

## Abstract

The Resource Description Framework (RDF), together with well-defined ontologies, significantly increases data interoperability and usability. The SPARQL query language was introduced to retrieve requested RDF data and to explore links between them. Among other useful features, SPARQL supports federated queries that combine multiple independent data source endpoints. This allows users to obtain insights that are not possible using only a single data source. Owing to all of these useful features, many biological and chemical databases present their data in RDF, and support SPARQL querying. In our project, we primary focused on PubChem, ChEMBL and ChEBI small-molecule datasets. These datasets are already being exported to RDF by their creators. However, none of them has an official and currently supported SPARQL endpoint. This omission makes it difficult to construct complex or federated queries that could access all of the datasets, thus underutilising the main advantage of the availability of RDF data. Our goal is to address this gap by integrating the datasets into one database called the Integrated Database of Small Molecules (IDSM) that will be accessible through a SPARQL endpoint. Beyond that, we will also focus on increasing mutual interoperability of the datasets. To realise the endpoint, we decided to implement an in-house developed SPARQL engine based on the PostgreSQL relational database for data storage. In our approach, data are stored in the traditional relational form, and the SPARQL engine translates incoming SPARQL queries into equivalent SQL queries. An important feature of the engine is that it optimises the resulting SQL queries. Together with optimisations performed by PostgreSQL, this allows efficient evaluations of SPARQL queries. The endpoint provides not only querying in the dataset, but also the compound substructure and similarity search supported by our Sachem project. Although the endpoint is accessible from an internet browser, it is mainly intended to be used for programmatic access by other services, for example as a part of federated queries. For regular users, we offer a rich web application called ChemWebRDF using the endpoint. The application is publicly available at https://idsm.elixir-czech.cz/chemweb/.

## Introduction

The role of information has become crucially important in many aspects of human activities, including in life science research. The size of life science datasets, their complexity, and their number have increased continuously over time. Due to this increasing size and complexity of datasets, the user effort that is needed to obtain relevant data increases too, especially when data are spread out over multiple heterogeneous datasets. There still does not exist a general solution for this issue. This is partly due to the fact that datasets are often exported in different formats. Another important reason is that datasets often use different ontologies, meaning one thing can be expressed in different ways in different datasets. What is worse, property names can collide, i.e., a certain property name can in fact express different properties in different datasets. Last but not least, the precise semantics of properties used in a dataset is often documented with insufficient accuracy.

**Fig. 1:**
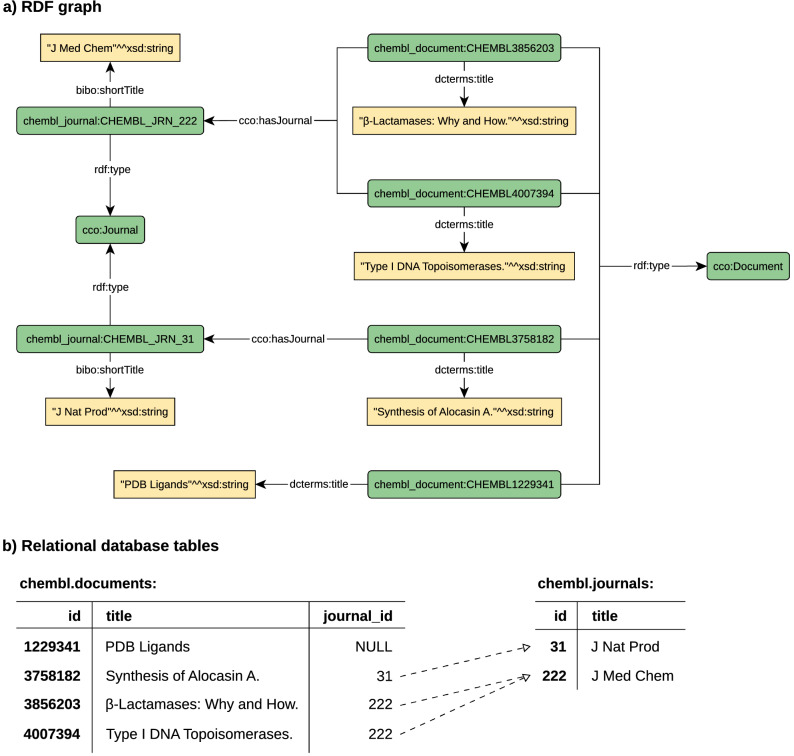
ChEMBL data example

**Fig. 2:**
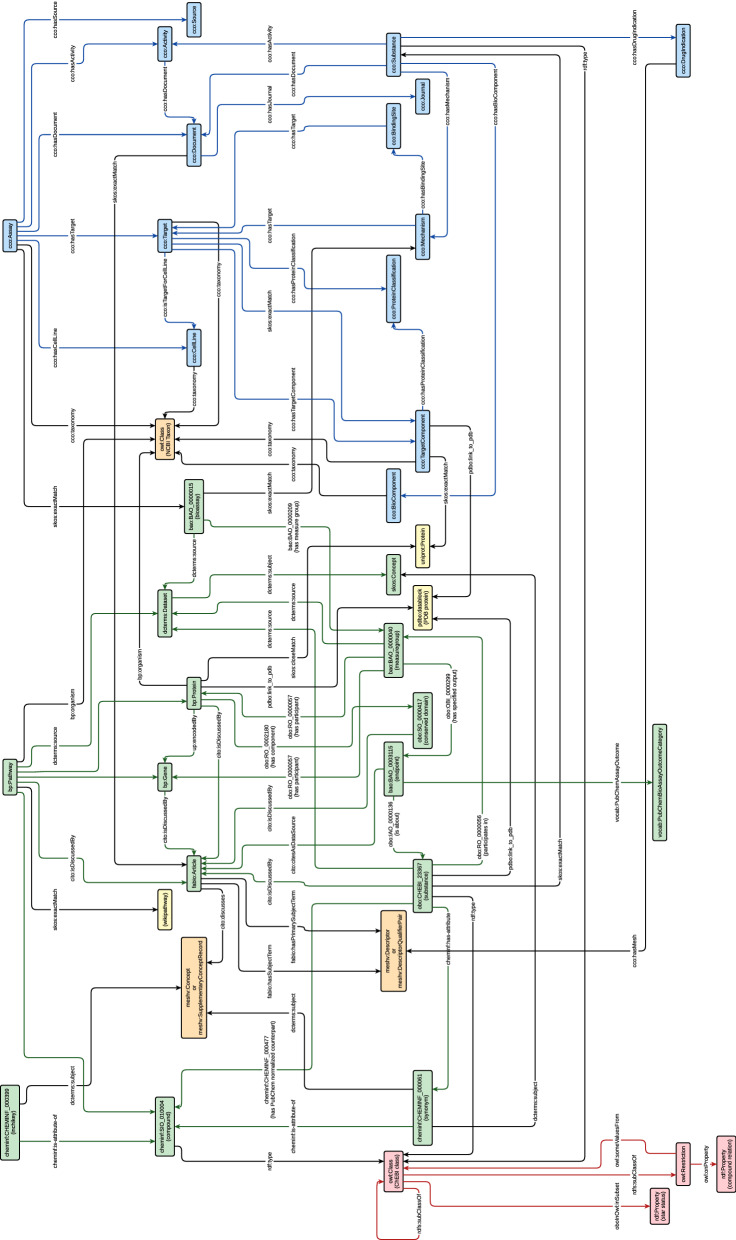
IDSM database schema

One of the approaches that can be used to improve the interoperability of datasets produced by heterogeneous providers is to adopt Linked Data principles [1]. According to these principles, things should be identified by uniform identifiers, connected with useful information accessible from the Internet in standard formats over the HTTP protocol and interlinked with other relevant things. The Semantic Web technologies were introduced to implement this approach. The main technology is the Resource Description Framework (RDF) [2, 3]. The framework is intended to represent information about resources where a resource is an abstraction of an entity in the world. In this framework, each piece of information is expressed as a subject-predicate-object triple denoting that the subject is related to the object, where the relation is denoted by the predicate. Although this basic concept is very simple, it is able to express very complex information as a set of such triples. A set of triples is often visualised as a connected graph with nodes representing subjects and objects and with labelled arcs representing predicates. An example of such an RDF graph can be seen in Fig. 1a. For comparison, the same information presented in the relational form is shown in Fig. 1b.

The RDF framework employs the Internationalised Resource Identifiers (IRIs) to identify resources [4]. The IRIs have a global meaning which makes interlinking between various datasets easy and straightforward. The predicates are also identified by IRIs, which avoids the situation where a certain property name has different meanings in different datasets. A set of IRIs used for describing resources related to an area of interest is called an RDF vocabulary. The RDF Schema (RDFS) [5] and Web Ontology Language (OWL) [6] have been introduced for the definition of RDF vocabularies and ontologies, which helps to define the precise semantics of data. The SPARQL query language was introduced in order to support querying RDF datasets [7]. One of its most important features is the federated query extension. It allows a SPARQL service to redirect a portion of a query to another SPARQL service, and to then combine obtained results with results from the rest of the query [8]. This highly increases interoperability, because one query allows users to gather information from different datasets. Each SPARQL service listens for requests at a specific IRI that is generally known as a SPARQL endpoint [9].

Several former (currently discontinued) pioneering projects decided to take advantage of the fact that many life science datasets are publicly available for download, and they have converted these datasets into RDF. Bio2RDF [10, 11] integrated data from some of the most popular public bioinformatics databases, and supported the conversion of primary resource entries into RDF on demand. Entries have been identified by IRIs in the generic form http://bio2rdf.org/namespace:id. Each namespace was associated with a JSP script connected to the appropriate primary resource in order to obtain information about an entity identified by the id used in an IRI, and to then return a result formatted in RDF. Chem2Bio2RDF [12] was focused on the creation of a single RDF repository that aggregated data from multiple chemogenomics repositories (including small molecule, target, gene, pathway and drug information). Similarly, Linked Life Data [13] was a platform converting public biomedical databases into RDF and accessing them through a single SPARQL endpoint. In addition to projects focused on converting and integrating multiple datasets, there were also projects focused on single datasets. For example, ChEMBL-RDF [14, 15] was focused on converting the ChEMBL dataset into RDF, and then linking it with other existing RDF datasets.

Some initiatives were established that focused on cooperation between various subjects that publish RDF datasets. Linking Open Drug Data (LODD) [16, 17] was a task force within the World Wide Web Consortium’s (W3C) Health Care, and Life Sciences Interest Group (HCLS IG) aimed to create life science RDF datasets provided by individual participants. The datasets should be linked with each other, as well as with datasets provided by other Linked Data projects. Open PHACTS [18] was a European initiative aimed to use Semantic Web technologies to develop a platform that improves collaboration between industry and public organisations focused on drug discovery.

At present, many important life science datasets offer their data directly in the RDF. These include UniProt (database of protein sequences and associated detailed annotation) [19], PubChem (open repository for chemical structures, biological activities and biomedical annotations) [20], ChEMBL (bioactivity database) [21, 22], ChEBI (database and ontology of chemical entities of biological interest) [23], neXtProt (human protein database) [24, 25], Rhea (database of expert-curated biochemical reactions) [26], PDBj (protein database maintained by the Protein Data Bank Japan, a member of the worldwide Protein Data Bank) [27], WikiPathways (database of biological pathways) [28], DisGeNET (database of gene-disease associations) [29, 30], and OMA (orthology database) [31].

A SPARQL endpoint for querying a dataset is often introduced together with the dataset. Some projects do not use their own SPARQL server but instead use a platform providing SPARQL endpoints for multiple datasets. The EBI RDF platform should coordinate RDF activities across the European Bioinformatics Institute (EBI), and it should also provide a SPARQL endpoint to querying resources available at the institute [32]. Unfortunately, the platform has currently no funding, meaning its support is sporadic at best. The National Bioscience Database Center (NBDC) RDF portal aims to promote Japanese database development groups in order to expose their datasets in RDF [33]. This portal offers SPARQL endpoints for these datasets and also, for example, for PubChem and UniProt.

When RDF began to be used in life sciences, newly created RDF datasets often used ad-hoc ontologies typically reflecting the original format of the datasets. However, such ontologies do not allow good interoperability between datasets. To address this issue, ontologies designed to be interoperable began to gain more ground. Such ontologies were not developed to be used in a particular dataset. Instead, they were developed to describe a selected domain independently of individual datasets. The ontology should be general enough to be usable in any dataset focused on the given domain. Usage of such ontologies significantly improves interoperability between datasets.

There are many ontologies focused on particular life science domains: BioAssay Ontology (BAO), describing chemical biology screening assays and their results for the purpose of categorising assays and data analysis [34]; Protein Ontology (PRO), defining protein-related entities and showing relationships between them [35]; Gene Ontology (GO), focused on functions of genes and gene products [36]; Medical Subject Headings (MeSH), used for indexing, searching and cataloguing of biomedical and health-related information [37]; Chemical Information Ontology (CHEMINF), specifying chemical information entities, e.g., chemical graphs and descriptors, algorithms and their software implementations, chemical data formats [38]; Semanticscience Integrated Ontology (SIO), offering classes and relations to describe and relate objects, processes and their attributes with specific extensions in the biomedical domain [39]; and the EDAM ontology, related to bioinformatics operations, types of data and identifiers, data formats and topics [40]. All these listed ontologies are accessible through NCBO BioPortal that provides access to a library of biomedical ontologies, their subsets and user comments [41].

Life science datasets also widely use various general-purpose ontologies. They include, for example, Dublin Core Metadata Initiative (DCMI) Terms focused on cross-domain resource description in order to achieve easier search and retrieval [42], FRBR-aligned Bibliographic Ontology (FaBiO) and Citation Typing Ontology (CiTO) designed for describing bibliographic resources and citations [43], and Simple Knowledge Organization System (SKOS) providing a data model and vocabulary for expressing knowledge organisation systems [44].

Several standards to describe the content of datasets have been introduced as well. To describe life science datasets, their versions and file formats in which the datasets are available, the W3C Semantic Web for Health Care and Life Sciences (HCLS) Interest Group introduced a community profile for describing datasets [45]. The profile is mainly based on more general vocabularies: the Data Catalog Vocabulary (DCAT) designed to facilitate interoperability between data catalogues [46] and the Vocabulary of Interlinked Datasets (VOID) aimed for expressing metadata about RDF datasets [47]. The SPARQL Service Description vocabulary has been introduced to describe SPARQL services [48].

Our project presented in this paper is focused on small-molecule datasets. The goal of the project is twofold: firstly to integrate various datasets into one database called the Integrated Database of Small Molecules (IDSM) accessible through a SPARQL endpoint, and secondly to improve mutual linking between the integrated datasets and their interoperability with other datasets that are referenced from these integrated datasets, when necessary. To enhance usability of the endpoint, the substructure and similarity search of compounds is also supported by utilisation of our Sachem project [49, 50]. We also focus on the development of a web application named ChemWebRDF that allows users to write SPARQL queries and to explore data in a user-friendly way. In the first stage, we decided to use only datasets having RDF representations introduced directly by data providers. This is very important for us at this stage, as we do not want to create an unauthorized RDF form of a dataset. There is a very good reason for this. Some datasets have been converted into RDF by different teams (for example, ChEMBL [11, 14, 15, 21] or MeSH [51]). Although another RDF representation of a dataset can be very useful for a specific purpose of use, experience shows that such fragmentation decreases the overall interoperability as an unwanted side effect. We are currently focused on the following datasets: PubChem, ChEMBL and ChEBI. These datasets are well established, and have their RDF representations created directly by their providers. The other useful thing is that these datasets are already interlinked, so they represent a good basis for the creation of a service that will integrate various small-molecule datasets. Moreover, none of these datasets currently has an official and supported SPARQL endpoint.

All three datasets are available for bulk download in RDF on their web sites. The PubChemRDF project additionally provides programmatic data access through RESTful interface that allows querying RDF triples [20]. However, only one triple pattern is allowed in a query submitted by this interface. Since the beginning of 2020, the PubChem RDF data have been accessible through the NBDC RDF portal. However, it has updated the data only once since then. The ChEMBL datasets was accessible through official Linked Open Data platform for EBI data [32]. Unfortunately, this platform is not currently supported, and it has not contained any ChEMBL data for a long time. As an unofficial replacement, the department of Bioinformatics (BiGCaT) at Maastricht University has recently started offering a SPARQL endpoint that mirrors the ChEMBL RDF dataset [52].

**Table 1:** Definitions of namespace prefixes

Prefix	Definition
rdf:	http://www.w3.org/1999/02/22-rdf-syntax-ns#
rdfs:	http://www.w3.org/2000/01/rdf-schema#
xsd:	http://www.w3.org/2001/XMLSchema#
owl:	http://www.w3.org/2002/07/owl#
bao:	http://www.bioassayontology.org/bao#
bibo:	http://purl.org/ontology/bibo/
bp:	http://www.biopax.org/release/biopax-level3.owl#
cco:	http://rdf.ebi.ac.uk/terms/chembl#
chebix:	http://purl.obolibrary.org/obo/chebi#
cito:	http://purl.org/spar/cito/
cv:	http://nextprot.org/rdf/terminology/
dcterms:	http://purl.org/dc/terms/
fabio:	http://purl.org/spar/fabio/
cheminf:	http://semanticscience.org/resource/
meshv:	http://id.nlm.nih.gov/mesh/vocab#
nextprot:	http://nextprot.org/rdf#
obo:	http://purl.obolibrary.org/obo/
oboInOwl:	http://www.geneontology.org/formats/oboInOwl#
pdbo:	http://rdf.wwpdb.org/schema/pdbx-v40.owl#
rh:	http://rdf.rhea-db.org/
sachem:	http://bioinfo.uochb.cas.cz/rdf/v1.0/sachem#
sio:	http://semanticscience.org/resource/
source:	http://rdf.ncbi.nlm.nih.gov/pubchem/source/
skos:	http://www.w3.org/2004/02/skos/core#
taxon:	http://purl.uniprot.org/taxonomy/
uniprot:	http://purl.uniprot.org/uniprot/
up:	http://www.uniprot.org/core/
vocab:	http://rdf.ncbi.nlm.nih.gov/pubchem/vocabulary#

## Implementation

For convenience, prefixed names are used instead of the full IRIs in the following sections that describe the schema of the IDSM database and its use cases. The definitions of namespace prefixes used in the paper are summarised in Table 1.

### Database schema

The RDF schema of the IDSM database reflects RDF schemas of selected datasets (i.e., PubChem, ChEMBL and ChEBI) as we integrate datasets already exported to RDF.

The PubChem dataset comprises three main parts: substances, compounds and bioassays. Substances are deposited into the dataset by chemical vendors (data sources). Different vendors can deposit the same chemical structure. Each deposited record is assigned a unique identifier. Substance records are standardised to compounds (class cheminf:SIO_010004) that represent a set of unique chemical structures. During this process, substances representing the same chemical entity are aggregated into a single compound, and links (predicate cheminf:CHEMINF_000477) between the substances and the corresponding compound are added. Screening data are deposited as bioassays (class bao:BAO_0000015). Biological assays are divided (predicate bao:BAO_0000209) into measure groups (class bao:BAO_0000040) containing (predicate obo:OBI_0000299) individual screening measurements represented by bioactivity endpoints (class bao:BAO_0003115). Measure groups can point at (predicate obo:RO_0000057) screened proteins (class bp:Protein) or genes (class bp:Gene). Each endpoint is linked (predicate obo:IAO_0000136) to the tested substance, as well as being linked (predicate vocab:PubChemAssayOutcome) to the screening outcome (class vocab:PubChemBioAssayOut-comeCategory). Endpoints can also refer (predicate cito:citesAsDataSource) to publication references (class fabio:Article) describing details of the measurements.

ChEMBL is also focused on biological activities, but it uses a different ontology to express bioassay experiments. However, both datasets share a similar basic concept. Only unique and standardised substances (class cco:Substance) are stored in the ChEMBL dataset. Biological assays (class cco:Assay) are not divided into measure groups, and they are directly linked (predicate cco:hasTarget) to screened targets (class cco:Target). Targets include proteins and their complexes, organism, cell-lines, tissues, etc. Targets are divided (predicate cco:hasTargetComponent) into molecular components (class cco:TargetComponent)—usually proteins. Individual measurements are represented by activities (class cco:Activity) that are linked (predicate cco:hasActivity) from tested substances, as well as from assays to which they belong. In addition, it also contains information about drug mechanisms of actions (class cco:Mechanism). Mechanisms are specified by text descriptions (predicate cco:mechanismDescription, predicate cco:mechanismActionType). They are referenced from substances (predicate cco:hasMechanism) having the given actions, and are linked (predicate cco:hasTarget) to targets of these drug mechanisms. Alternatively, they are linked (predicate cco:hasBindingSite) to binding sites (class cco:BindingSite) on specified (predicate cco:hasTarget) targets. Documents (class cco:Document) with supporting information should be linked (predicate cco:hasDocument) from substances, assays and activities.

Unlike the previous two datasets, the ChEBI dataset contains no assay data, and it is solely focused on creating a hierarchical classification of compounds. In ChEBI, each molecular entity is expressed as a class. Relations between ChEBI classes are then described in RDF using the Web Ontology Language (OWL). Hierarchy is denoted by the standard rdfs:subClassOf predicate. Other relations between ChEBI classes are described indirectly by using OWL restrictions. For example, ChEBI class X is a conjugate acid of ChEBI class Y if and only if, for each compound x from X, there is a compound y from Y, such that x is the conjugate acid of y. This fact is expressed in OWL in the following form: X is a subclass of the class of all individuals for which at least one value of the chebix:is_conjugate_acid_of predicate is an instance of the class Y.

In addition to these three small-molecule datasets, the IDSM database also integrates ontologies that are used by these three datasets, including extensive MeSH [37] and NCBITaxon [53] ontologies.

A simplified summary of the IDSM database RDF schema is shown as a graph in Fig. 2. Graph nodes represent resource classes, whereas arcs represent predicates used by the database to capture relation between resources. Each arc is oriented from its predicate domain to its predicate range. Nodes and arcs are labelled by IRIs of appropriate resource classes and predicates, respectively. Labels in parentheses were added to the IRIs where necessary for greater clarity. The colours of nodes are used to distinguish datasets. Green, blue and red colours are used to denote resources from PubChem, ChEMBL and ChEBI datasets, respectively. The orange colour denotes integrated ontologies, and yellow denotes other referenced datasets.

### Dataset enhancement

Datasets can contain additional data that are not exported to RDF by datasets creators. Since some parts of the additional data can be useful for better interoperability, we decided to integrate these parts into the IDSM database.

We enhanced the IDSM database with all compound structures included in the selected small-molecule datasets. The structures are expressed in the MDL Molfile format. We present these structures in the same way that is used by PubChem to express the linkage from compound to the chemical descriptor resources (e.g., InChI, SMILES, ...). Similarly to other chemical descriptor resources, the MDL Molfile values are linked from these resources by the sio:has-value predicate, and these resources are linked to corresponding compounds by the sio:is-attribute-of predicate. The only difference is that these resources are typed as sio:SIO_011120 (molecular structure file).

For PubChem bioassays, only their titles and sources are exported to RDF. Moreover, they are not exported for some of the bioassays. To increase the usability of the IDSM database, we decided to load the titles, source links and also bioassay specifications (including descriptions, protocols, and comments) for all PubChem bioassays. For better interoperability, we decided to employ standard BioAssay Ontology classes to export the bioassay specifications into RDF instead of creating new ones. We use the bao:BAO_0000522, bao:BAO_0000523 and bao:BAO_0000525 classes to denote bioassay descriptions, protocols and comments, respectively.

We also added some triples that enhance the mutual integration of selected small-molecule datasets, even though they use different ontologies. The PubChem dataset integrates data coming from various sources, including ChEMBL. If two resources represent the same thing, the skos:exactMatch predicate can be used to map one resource to the other. And exactly this predicate is used by PubChem to establish links between PubChem substances coming from ChEMBL and corresponding substances in the ChEMBL dataset. Although PubChem integrates ChEMBL assays as well, links between the integrated bioassays and their original ones are not exported to RDF. Nevertheless, the origins of PubChem assays are stored in primary PubChem data. We decided to use these data to add appropriate links between PubChem bioassays and their corresponding ChEMBL ones. Not only does PubChem integrate bioassays from ChEMBL, but some assays are also integrated from PubChem to ChEMBL. The ChEMBL dataset uses dedicated resources to describe links between them. For better interoperability, we also enhanced the IDSM database by using the skos:exactMatch predicate in this case.

Furthermore, each PubChem reference (class fabio:Article) contains a PubMed identifier (PMID) as a part of its IRI. Similarly, documents (class cco:Document) coming from ChEMBL are linked (predicate bibo:pmid) with their PubMed identifiers. Based on this information, we established skos:exactMatch mappings between instances sharing the same PubMed identifiers.

In order to link PubChem substances and compounds with ChEBI compounds, the rdf:type predicate is used in the PubChem dataset. This reflects the fact that ChEBI compounds are defined as classes. We used information stored in the ChEMBL datasets to employ the same kind of linking between ChEMBL substances and ChEBI compounds.

### Interoperability enhancements

Because IRIs have a global meaning, it is theoretically very simple to reference a resource described in one RDF dataset from another RDF dataset—the dataset just uses the resource IRI to reference it. It presents an unambiguous and effective way to link resources coming from different datasets. Issues arise when there is no general agreement on which IRIs should be used to identify resources from a dataset. This typically happens in cases where there is no official and widely used RDF representation of the dataset. In such a situation, it is possible to use the Identifiers.org Resolution Service [54], for example. This service allows the use of IRIs in the general form: https://identifiers.org/prefix:ID, where prefix is a registered identifier of a dataset, and id is a local accession identifier of a resource in the dataset. However, other approaches are also possible. This can lead to situations where two datasets use different ways to reference resources from another dataset. In such a situation, interoperability between these datasets is limited.

We identified several such issues related to referencing that we solved in order to enhance interoperability between datasets. In the case where the integrated datasets used different (or inconvenient) ways to reference another dataset, we enhanced them so that they all use the same common way to reference the dataset. However, we left the original ways unaffected and still present in the datasets in order not to break interoperability with other services that may depend on this usage.

To reference NCBI organismal taxa [55], the Identifiers.org service is used by ChEMBL and PubChem datasets. Because there is no official RDF version of the dataset, we employed the OBO/OWL translation of the NCBI organismal taxonomy provided by the OBO Foundry [53]. Unfortunately, this translation uses different IRIs for taxa. To address this issue, we improved ChEMBL and PubChem datasets to use these IRIs as well.

The ChEMBL dataset uses the Identifiers.org service to also link the MeSH ontology. Nevertheless, the authorised RDF version of the MeSH ontology uses different IRIs to identify headings. Thus, the appropriate IRIs have been added to the ChEMBL dataset in this case as well. The PubChem already uses the authorised IRIs, therefore no changes were needed.

The Identifiers.org service is used also by the ChEMBL dataset to link the PDB dataset. As for ChEMBL compounds and assays, dedicated resources are used to reference ChEMBL target components to PDB proteins. For better interoperability, links using the official ontology provided by Protein Data Bank Japan have been added to the ChEMBL dataset. Although the PubChem already uses the official ontology, it unfortunately uses an outdated version. Hence, links using the up-to-date ontology have been added to the PubChem dataset as well.

### SPARQL engine

To run a robust SPARQL endpoint, we experimented with several SPARQL engines during recent years, but none of them was fully sufficient for our purposes in terms of extensibility, reliability or efficiency. For this reason, we decided to implement an in-house SPARQL engine that supports the SPARQL 1.1 specification. For reliability, we use the well-established PostgreSQL relational database [56] for data storage. In our approach, datasets are stored in a relational database using optimised database schemas designed specifically for the given datasets. Special mappings from relational database schemas to RDF datasets are introduced in order to present the stored data in the RDF form. These mappings are then used to translate SPARQL queries operating on RDF data to equivalent SQL queries operating on relational data. The approach is similar to the Linked Data Views from the Virtuoso database [57], to the D2RQ mapping language [58] and to the R2RML mapping language [59] implemented, for example, in the Oracle Database [60]. In our approach, each dataset mapping consists of two basic parts:

1. *IRI mappings describe mappings between IRIs and SQL datatypes.* In RDF, all resources are identified by IRIs. However, it is very inefficient to store IRIs directly to a relational database and to use them to identify database records. For this reason, IRIs are mapped into more efficient SQL types. More precisely, all IRIs used to identify resources in the dataset are divided into disjunct classes, and for each IRI class there is a bijection defined between the class and SQL type(s). Values of these SQL types then can be used to identify resources stored in a relational database unambiguously and efficiently.
2. *Quad Mappings describe mappings between rows of tables and RDF triples.* RDF datasets are organised into collections of RDF graphs that contain a set of RDF triples. Thus, RDF datasets can be viewed as a set of RDF quadruplets. In order to map the rows of a specified table into quadruplets, for each term in a quadruplet mapping (i.e., for subject, predicate, object and graph) it is specified from which column (or columns) the term is generated. If a term is an IRI, the information about its IRI class is also included.

Although a dataset mapping describes a conversion from a database schema of a relational database to an RDF dataset, the conversion is never performed directly, and the RDF dataset is never materialised. The real purpose is the opposite. The mapping is used to convert incoming SPARQL queries operated on the RDF dataset to SQL queries operated on the target relational database. For each triple pattern occurring in a SPARQL query, it is determined which quad mappings generate triples that can be matched by the pattern. These mappings are then used to generate simple SQL statements that select requested data specified by corresponding patterns. These SQL statements are joined into a SQL query respecting SPARQL semantics. To achieve maximum effectiveness, database schema constraints (e.g., primary and foreign keys), together with information about IRI classes, are used during the translation in order to produce optimised SQL queries.

We have used a small portion of the ChEMBL data shown in Fig. 1 to demonstrate how the IDSM SPARQL engine works. In Fig. 1a, data are expressed in the form of an RDF graph. This particular piece of data describes information about four documents and two journals. Resources representing journals are typed as cco:Journal, and have been assigned short titles via the predicate bibo:shortTitle. Similarly, resources representing documents are typed as cco:Document, and have been assigned titles via the predicate dcterms:title. If a document was published in a journal, it is denoted by the predicate cco:hasJournal.

As shown in Fig. 1b, the same information can be expressed in a relational database by two tables. Table chembl.journals and table chembl.documents represent information about journals and documents, respectively. In both tables, each table row represents one resource. Individual resources are identified by integer values stored in appropriate id columns. These values were obtained from the resource IRIs by cutting off their non-numeric fixed parts. Resource titles are stored in relevant columns. The journal_id column referencing table chembl.journals is used to express links between document and journals.

**Fig. 3:**
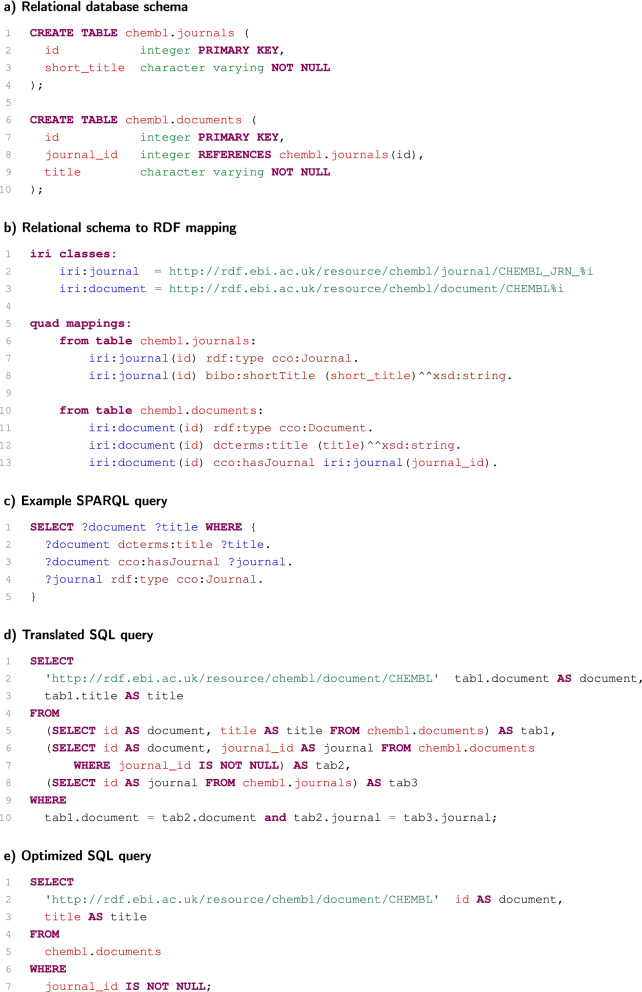
Basic idea of mapping and SPARQL query translation

**Fig. 4:**
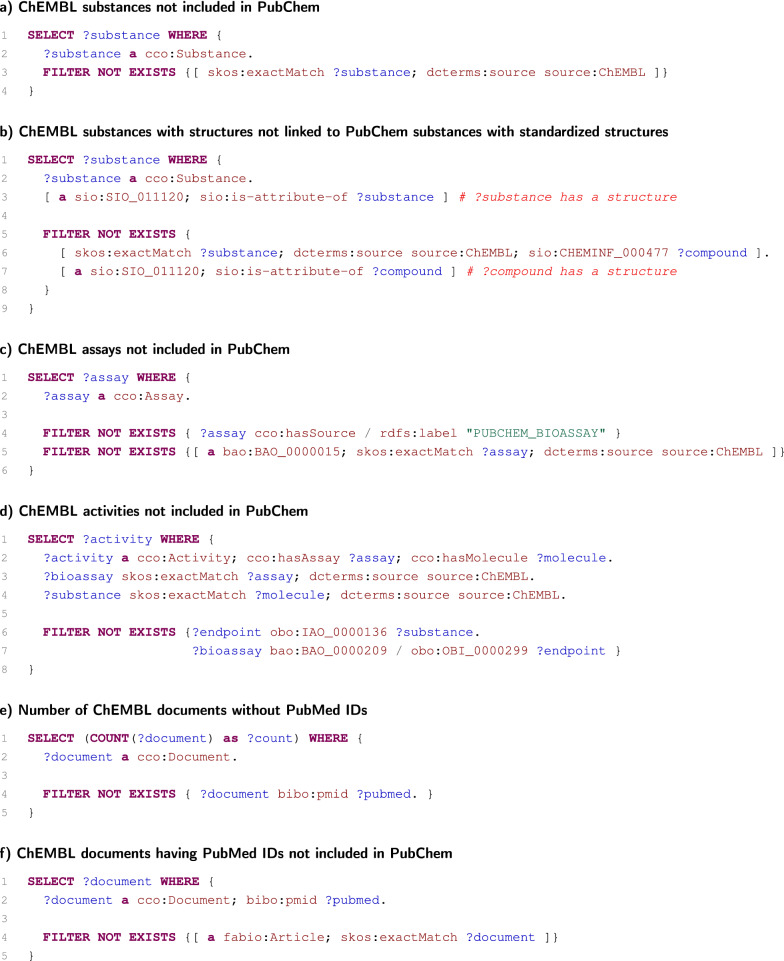
SPARQL queries to observe how well is ChEMBL included by PubChem

The example relational database (Fig. 1b) can be simply mapped into RDF (Fig. 1a) and queried by SPARQL, as demonstrated in Fig. 3. The schema of the tables used by the example data is shown in Fig. 3a. The mapping in Fig. 3b is utilised to map this relational schema into RDF. Firstly, two IRI classes are defined, one for the journal IRIs and one for the document IRIs. The classes describe how integer identifiers are converted into IRIs by adding appropriate fixed prefixes. Secondly, a set of quad mapping is defined for each table. For example, quad mapping ‘iri:document(id) dcterms:title (title)^^xsd:string’ for table chembl.documents denotes that each row of the table is mapped on the triple having the subject generated from column id by IRI class iri:document, the predicate bibo:shortTitle, and the object with a value from column short_title typed as xsd:string. The triples are stored in the default RDF graph, and therefore the graph declaration is omitted from the quad mapping. For all quad mappings, there is the following condition: a table row is used for a quad mapping only if all required values are not null.

The introduced mapping is used to translate a SPARQL query into an SQL query that generates an equivalent result. Quad mappings generating triples that can be matched by a triple pattern are selected for each triple pattern in the SPARQL query. These quad mappings are then used to generate SQL SELECT clauses to obtain requested data. The clauses are joined according to the SPARQL semantics to obtain the final result. For example, triple pattern ‘?document dcterms:title ?title’ can match triples generated by the quad mapping mentioned above. This quad mapping is then used to generate the appropriate SQL SELECT clause retrieving documents and their titles.

A full translation of the example SPARQL query from Fig. 3c is shown in Fig. 3d. However, this translation can be optimised if the database schema in Fig. 3a is taken into account. Tables tab1 and tab2 are derived from the same table, and they are joined according to the table primary key. Thus, these two SQL SELECT clauses can be merged. Moreover, table tab3 derived from table chembl.journals is joined with table tab2 according to the foreign key journal_id that references the primary key in this table. For this reason, table tab3 can be eliminated in this case, because no additional information from table tab3 is required. Fig. 3e shows the final SQL query obtained after the optimisations are performed.

### Loading datasets

In spite of the fact that the selected datasets are exported to RDF, these data cannot be used to load datasets into the IDSM database without additional effort because our engine is not a native triple store. In general, three steps are needed to support a dataset due to the fact that a relational database is used to store data:

1. *Design an optimised relational schema.* Although it is possible to define one table separately for each set of triples sharing the same predicate and linking resources of same classes, it is more efficient to use one table to store information coming from multiple kinds of triples (i.e., using different predicates). For example, the fact that a substance was tested in a measure group of a bioassay with a particular outcome is expressed by four triples in the PubChemRDF dataset. However, because all these triples are very closely related and none of them exists independently from the others, the same information can be expressed by one row of a table.
2. *Design mappings from the schema back into RDF.* This step is closely related to the previous step and represents the inverse process. Each decision in the first step to represent a set of triples by a table leads to quad mappings in which this table is used to generate identical set of triples.
3. *Implement a loader that converts RDF data and stores them into the database.* A particular way of loading data depends on formats that are used to export the selected dataset.

Although some datasets are also exported as SQL tables dump, and although the engine is general enough to employ these dumps, we prefer to load data that were exported to RDF rather than SQL dumps. Although these dumps allow loading data into a database to be a simple process, they offer no special support to perform updates, which are required to ensure that the IDSM database remains up-to-date when a new version of a dataset is released.

We have implemented several loaders for loading RDF data into the IDSM database. Each loader is specially designed to load some part of data from a particular dataset. In general, loaders have to support various RDF serialisation formats (e.g., RDF/XML format [61] or Turtle format [62]). There are two basic approaches depending on the complexity of the loaded data. If a file contains only triples sharing the same predicate, a simple loader processing a stream of triples can be employed to extract and to store the requested data. If data stored in a file are more complex, a loader employs the Apache Jena [63] to load the file into an in-memory RDF model. The model is then queried by the Jena SPARQL engine to extract the requested data. Similarly, if data are stored in a general XML file (e.g., PubChem bioassays), the file is loaded into memory as the Document Object Model (DOM), and the XML Path Language (XPath) [64] is used to extract requested data from this model. In all three cases, the extracted data are compared with data already stored in the appropriate database tables, and requested updates are performed. Data are updated in a single database transaction. This way, an update can be performed automatically, and therefore datasets are never presented in an inconsistent state.

All PubChem data available in RDF have been loaded into IDSM except compound similarities (predicates cheminf:CHEMINF_000482 and cheminf:CHEMINF_000483).

The largest of the loaded datasets utilized by IDSM is PubChem, containing almost 300 million substances, approximately 100 million compounds and more than one million bioassays. The ChEMBL dataset contains approximately 2 million molecules and more than one million assays. And finally, ChEBI describes more than 100 thousand entities. Current exact numbers are on the project web site.

### User interface

The SPARQL endpoint of the service is https://idsm.elixir-czech.cz/sparql/endpoint/idsm. Although the endpoint is accessible from an internet browser, it is mainly intended to be used for programmatic access by other services, for example, as a part of federated queries. For regular users, we offer the ChemWebRDF web application using the endpoint and located at https://idsm.elixir-czech.cz/chemweb/. The application helps users to edit a SPARQL query, it presents query results in a user-friendly way as retrieved resources are displayed together with additional information, and it allows users to walk through results in order to discover other relations. The application is based on our older approach [65], except that it uses the new SPARQL engine.

The user interface of the application is divided into three parts. The left part contains the SPARQL query editor. The query result is visualised in the central part of the application. And finally, the right part of the application is used to visualise details about the selected resource.

The query editor is based on the third-party CodeMirror component [66] that allows SPARQL syntax highlighting and auto-completion of predicates used in the IDSM database. The users can create a SPARQL query from scratch, or they can load a predefined query example that can be modified further. These examples were adopted from PubChemRDF use cases [67]. Another possibility is to use the application query wizard that allows users to generate queries searching for compounds, bioassays, participants (proteins or genes) or their combinations.

The result of a submitted query is shown as a result table in the central part of the application interface. Each variable used in the select clause of the query is represented by one column. Individual solution mappings forming single results are represented by rows. To present the result in a user-friendly way, a resource in the database can be associated with an item template written in the Velocity Template Language [68]. If a variable is bound to a resource with an assigned item template, the item template is then used to visualise the appropriate table cell. Otherwise, the value itself is used as the cell content. The visualisation typically contains the resource label that is separately searched in the IDSM database by a SPARQL query specified in the template.

Detailed information about a selected resource is provided on the right side of the application. The resource can be selected either by clicking on the appropriate cell in the result table or by typing its IRI directly. The Details tab is used for a detail visualisation of a supported resource that has been assigned a page template. If details about the resource are requested, the application uses the appropriate page template to generate details about the resource. The Properties tab is used to display all relations between the selected resource and other resources or values. It uses the given resource as a subject, and shows all predicates (properties) and objects (values) for which triples subject-predicate-object are stored in the IDSM database. There is also a tab with the application manual which contains more detailed information about the usage of the application.

## Results and discussion

Based on the data loaded into the IDSM database and exposed as the SPARQL service, we would like to discuss the two following subjects. First, we will discuss the quality of interlinking between the loaded datasets. Secondly, we will show how the service can be used to solve complex cases.

Because all prefixes used in the queries are predefined in our engine, we omit their definitions from the queries to make them shorter. As all triples are included in the IDSM database default graph, we also omit FROM clauses specifying queried graphs.

### Interconnectedness of PubChem and ChEMBL datasets

Both the PubChem dataset and the ChEMBL dataset store results of biological assays. Moreover, as it has already been stated, the PubChem dataset includes and links data from ChEMBL (and to a lesser extent, vice-versa). We take advantage of the fact that both datasets are loaded in one SPARQL service, and we employ a set of SPARQL queries to examine how thoroughly the data from ChEMBL are included in the PubChem datasets. The SPARQL queries used for the examination are listed in Fig. 4, and they were evaluated against ChEMBL 27 and PubChem 2020-12-13.

At first, we checked whether all ChEMBL substances are included in PubChem and denoted as coming from ChEMBL (Fig. 4a). Because substance can be deposited into PubChem without structures (which, however, are needed to standardise substances on PubChem compounds), we also checked whether all ChEMBL substances having structures are standardised to PubChem compounds having structures (Fig. 4b). In this case, we observed that there are 92 ChEMBL substances that do not satisfy this condition.

As in the case of substances, we checked whether all ChEMBL assays (not coming from PubChem) are included in PubChem as PubChem bioassays denoted as coming from ChEMBL (Fig. 4c). In this case, we found 6 assays that are lost in PubChem. In addition, for ChEMBL assays included in PubChem, we also determined that all ChEMBL activities of these assays are included in PubChem as endpoints (Fig. 4d).

Finally, we focused on documents as they are also crosslinked between both datasets. There are currently 8059 documents in ChEMBL that have not been assigned PubMed IDs (Fig. 4e). Unfortunately, these documents cannot be included in PubChem as it uses PubMed IDs to identify documents. Nevertheless, these documents represent only about one tenth of all documents in ChEMBL. As we observed, the remaining ChEMBL documents are fully included in PubChem (Fig. 4f).

As it was demonstrated, ChEMBL substances and assays are properly included in PubChem. Thus, PubChem provides a different view of these data, as it employs different ontologies compared to ChEMBL. Nevertheless, loading ChEMBL into our database cannot be considered as redundant, since there are ChEMBL entities that are not included in PubChem (e.g., drug mechanism).

### Use cases

**Fig. 5:**
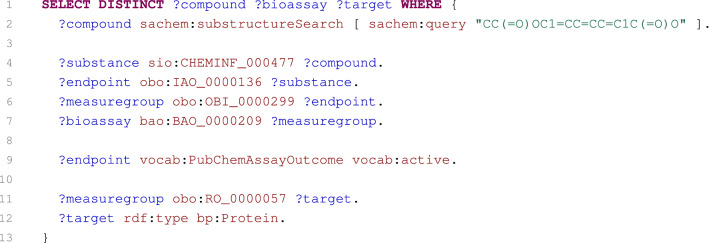
SPARQL query selecting aspirin-like compounds active in bioassays having protein targets

**Fig. 6:**
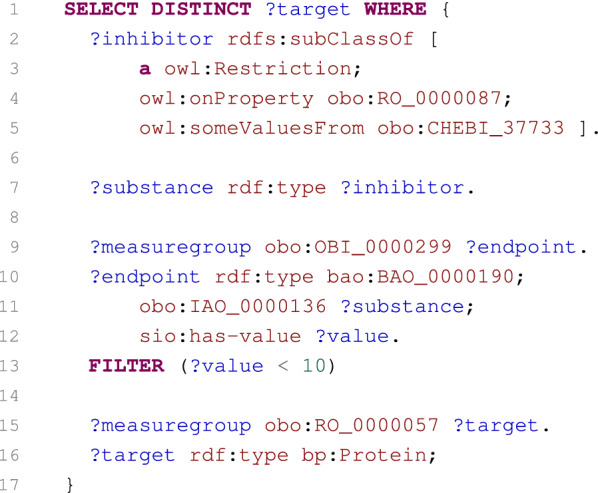
SPARQL query selecting proteins inhibited by cholinesterase inhibitors

**Fig. 7:**
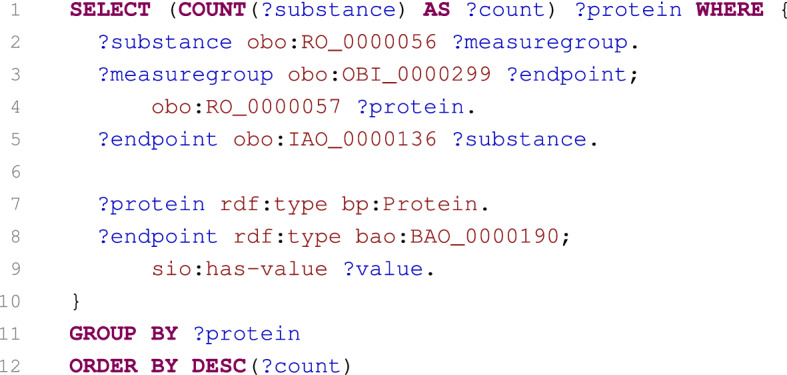
SPARQL query summarising numbers of substances tested by protein targets

**Fig. 8:**
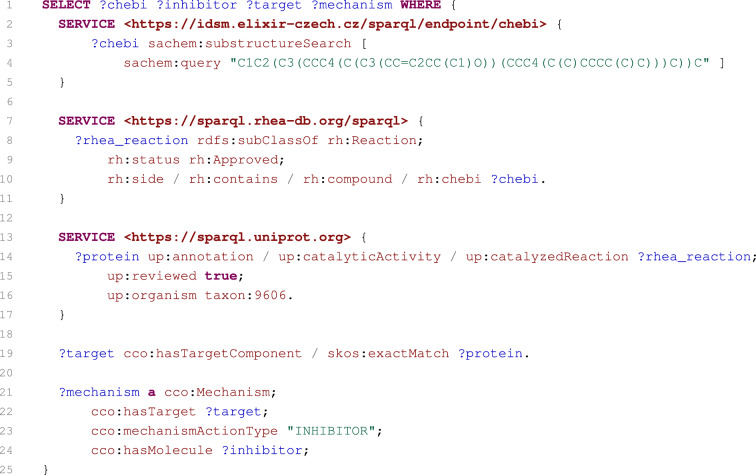
SPARQL query selecting inhibitors of proteins catalysing reactions of cholesterol or its derivatives

**Fig. 9:**
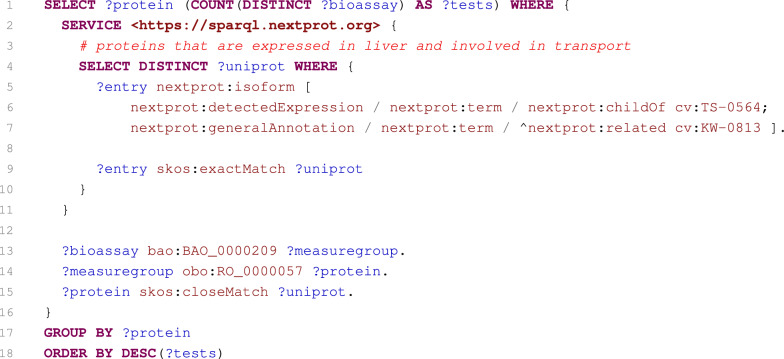
SPARQL query computing the numbers of bioassays with proteins expressed in the liver and involved in transport

To demonstrate the usefulness of our service, we present several use cases that together employ all loaded datasets (i.e., PubChem, ChEMBL, and ChEBI). SPARQL queries solving the individual cases are shown in Figs. 5, 6, 7, 8 and 9. It should also be noted that these queries do not explicitly load additional data about requested resources (e.g., their names), as these data are loaded automatically by our web interface that can be used to submit the presented queries.

#### Case 1: Aspirin-like compounds active in bioassays having protein targets

The first case is an example of a SPARQL query (Fig. 5) generated by the web application wizard. The wizard is able to generate various queries selecting interlinked compounds, bioassays and their targets from PubChem according to specified constraints. In this case, the query identifies PubChem compounds that contain acetylsalicylic acid as their substructure and that are active in bioassays containing protein targets. The corresponding compounds, bioassays and their targets are then returned as the query result.

To obtain compounds according to their structures, the integrated Sachem-based search of PubChem compounds is used (line 2). Because PubChem compounds represent a standardised set of chemical structures that are not directly linked with bioassays measurements, the obtained compounds are used to retrieve substances deposited by vendors (line 4). For these substances, relating endpoints, their measure groups and bioassays are selected (lines 5–7). The endpoints are constrained to select only active endpoints (line 9), and only protein targets of the measure groups are taken into account (lines 11–12).

Although the query may look slightly complicated as it employs a nontrivial number of triple patterns to describe relatively simple task, the SPARQL engine is optimised for such types of queries. Therefore, the queries can be satisfactorily evaluated.

#### Case 2: Proteins inhibited by cholinesterase inhibitors

The next case was adopted from the PubChemRDF site and illustrates how to select protein targets that are inhibited by cholinesterase inhibitors with an IC50 less than 10 $\upmu$M [67]. The cholinesterase inhibitors are selected from the ChEBI dataset, while the rest of data is selected from the PubChem dataset. Hence, the case simply demonstrates how the interconnection of PubChem and ChEBI can be employed to obtain requested information.

The corresponding SPARQL query is listed in Fig. 6. The first part of the query (lines 2–5) selects all ChEBI classes annotated to have the cholinesterase inhibitor role that is identified by the ChEBI:37733 identifier. Based on these classes, the relevant PubChem substances are obtained (line 7). Consequently, measure groups and their endpoints related to the substances and having an IC50 less than 10 $\upmu$M are identified (lines 9–13). Finally, protein targets of the endpoints are identified (lines 15–16) and returned as the query result.

#### Case 3: Summarise numbers of substances tested by protein targets

The third case is shown in Fig. 7 and it is also adopted from the PubChemRDF site [67]. It computes the total number of substances quantitatively tested (IC50) against each protein target.

The core of the query (lines 2–5) specifies linking between substances and their endpoints belonging to measure groups having protein targets (line 7), whereas only IC50 endpoints (line 8) associated with measured values (line 9) are taken into account. Selected resources are then grouped according to the proteins (line 11), total numbers of tested substances are calculated (line 1), and these numbers are subsequently used to sort the final results (line 12).

We include this case to demonstrate the ability of our SPARQL engine. On the PubChemRDF site, it is noted that the query may be time-consuming as it computes aggregate values for solutions retrieved by a conjunction of seven SPARQL patterns. Nevertheless, in our approach the query is transformed into a database execution plan where aggregates are computed from two efficient SQL joins of three tables: a table storing relations between substances, measure groups and endpoints; a table capturing protein targets of measure groups; and finally a table containing the measured values of endpoints. This allows the result to be obtained very quickly (in the order of tens of seconds).

It is interesting to note that the pattern on line 2 is redundant. This is due to the fact that if a substance belongs to an endpoint (line 5), and this endpoint belongs to a measure group (line 3), then it is satisfied that the substance belongs to the measure group (line 2). Nevertheless, the native triple stores have no information about this dependency between patterns, so they have to evaluate this pattern even if it is unnecessary. In contrast, our engines use the same SQL table to describe relations between substances, measure groups and endpoints, so this redundant pattern is eliminated during the translation of the query.

#### Case 4: Inhibitors of proteins catalysing reactions of cholesterol or its derivatives

The following case demonstrates the interoperability of the presented SPARQL service with other SPARQL services. The case extends an example available on the Rhea SPARQL endpoint site [69]. The original example retrieves human proteins that catalyse Rhea reactions involving cholesterol or its derivatives. This example already uses other SPARQL services to obtain requested data. We extend this example to obtain inhibitors of retrieved catalysts.

The final SPARQL query solving the extended task is shown in Fig. 8. Because the Rhea reaction database is built on the ChEBI, the IDSM/ChEBI service indexing ChEBI compounds is used first to obtain ChEBI compounds containing cholesterol as their substructure (lines 2–5). These compounds are then utilised by the Rhea service to identify reactions in which the compounds are involved (lines 7–11).

Since UniProt is interlinked with Rhea reactions, the UniProt SPARQL service [70] is then used to retrieve human proteins that catalyse identified reactions (lines 13–17). And finally, our service is used to select ChEMBL substances that are annotated as inhibitors of the selected proteins (lines 19–24).

This case perfectly demonstrates how it is possible to obtain useful and complex information spread out across several sources. Four independent SPARQL services (each of them focused on different topic) have been interconnected by the query to solve the complex task.

#### Case 5: Numbers of bioassays with proteins expressed in liver and involved in transport

The last case shows the interoperability of our service with another SPARQL service. The human protein database neXtProt contains many SPARQL examples on its site [71]. We chose an example that selects proteins expressed in the liver and involved in transport, and we extended it to obtain the numbers of PubChem bioassays in which the individual proteins have been tested.

At the beginning of the query (Fig. 9), the neXtProt service is employed to retrieve requested proteins (lines 2–11). Because PubChem is not directly linked with the neXtProt datasets, the UniProt identifiers of the proteins is also retrieved (line 9). Based on these UniProt identifiers, PubChem bioassays involving these proteins as their targets are identified (lines 13–15). Results are then aggregated according to proteins (line 17), and the number of bioassays is computed for each of the proteins (line 1). Query results are ordered according to these numbers of bioassays in descending order (line 18).

This case well demonstrates that collaboration of the services is possible, even if they are not directly linked to each other. In this particular case, it is sufficient that both datasets are interlinked with UniProt proteins.

## Conclusion

The paper introduced the Integrated Database of Small Molecules (IDSM) that is available as a SPARQL service based on an in-house SPARQL engine. This SPARQL service supports querying in the selected RDF small-molecule datasets, which include PubChem, ChEMBL and ChEBI. These datasets have been extended to improve their mutual interlinking. The usefulness of the service has been demonstrated with several examples that focused on the simultaneous use of multiple datasets supported by the service, as well as with examples focused on the interoperability between the service and other SPARQL services. It showed that the presented service can be used to solve complex tasks to obtain information spread out across several datasets. The SPARQL endpoint of the service is https://idsm.elixir-czech.cz/sparql/endpoint/idsm. A rich web application supporting querying in a user-friendly way has been introduced as well. The application allows writing queries in a SPARQL query editor, and it uses a template-based approach to present query results together with other relevant data. This makes SPARQL querying more convenient for users. The application is available at https://idsm.elixir-czech.cz/chemweb/.

## Data Availability

All datasets used are publicly accessible on their web sites. The service is public and freely available. Project source codes and installation notes are available in repositories chemweb, pgsparql, sachem, loaders and notes at https://bioinfo.uochb.cas.cz/gitlab/chemdb.
